# Governance for Social Purpose: Negotiating Complex Governance Practice

**DOI:** 10.3389/fpsyg.2020.579307

**Published:** 2020-09-23

**Authors:** Brigid Jan Carroll, Christa Fouche, Jennifer Curtin

**Affiliations:** ^1^Department of Management and International Business, Faculty of Business and Economics, University of Auckland, Auckland, New Zealand; ^2^School of Counselling, Human Services and Social Work, Faculty of Education and Social Work, University of Auckland, Auckland, New Zealand; ^3^School of Social Sciences, Faculty of Arts, University of Auckland, Auckland, New Zealand

**Keywords:** governance, paradox, indigenous, adaptive, health

## Abstract

Social purpose initiatives rarely take place in only one sector or policy domain. They are likely to cross sector, community, local, and national interests and, in so doing, require alternative governance arrangements that are responsive and sustainable. This article focuses on the process of forging such governance processes drawing on a case study characterized by complex cross-sector demands. The subject of the case study is a paradigm-breaking primary health and well-being initiative for a region of New Zealand with longstanding healthcare challenges, but contemporary possibilities. We were invited by the creators of this initiative to record and reflect on the challenges and successes and, from this, to identify what might be possible for future innovations. In doing so, we draw on the adaptive governance literature to frame the governance challenges and offer five paradoxes requiring collective navigation. We conclude with a series of recommendations on how such paradoxes are navigated for those needing to build governance practice in innovative social purpose initiatives and recognize the importance of engaging with indigenous scholarship in future analyses.

## Introduction

The focus of this inquiry is on collaborative, grassroots or so-called “adaptive governance” in the pursuit of social purpose outcomes which tend to cross sector, community, local, and national interests and, in so doing, require new governance arrangements to be forged and sustained. Drawing on a specific case from Aotearoa New Zealand, this article explores the possibility of configuring governance for social purpose in a way that brings community, public sector and iwi (indigenous tribal entities) groups together. We recognize that, for the latter, collective, deliberative, and decentralized decision-making has a long cultural history, but is less embedded for those immersed in a colonial-informed bureaucratic system of government. Globally, and most certainly also in Aotearoa New Zealand, many non-indigenous policy makers and governance agencies are in the early stages of understanding collective, decentralized decision-making but are becoming increasingly aware of its potential to address intractable and cross-sector challenges. The focus of this article is on a particular instance of configuring governance for social purposes driven by a collective of community groups, public sector agencies, and iwi. For non-indigenous researchers and policy practitioners, the early stages of this process often lack visibility as they can occur after initial discussions have started but before any contracting, formalized institutional agreements or funding bids occur. They also mark the phase where initiatives are at their most pliable, uncertain, formless, and vulnerable. Careful navigating of such uncertainty up to the point where decision-making processes, structure and legitimation can be established is critical. The guiding purpose for this analysis, therefore, is to provide points of reference for those in the social purpose sector who might have begun or joined such initiatives. These points of reference can help to navigate this early phase, to “read” the cues and signs of movement, and gain a governance footing amidst genuine ambiguity, complexity and confusion.

We draw on an initiative we call *Grassroots Health*. *Grassroots Health* represents a ground-breaking and transformational social good initiative in its scope, purpose, inclusion, and process. Its purpose was to shift the direct responsibility and leadership of the health of one of New Zealand’s most economically challenged regions to the community itself. Key to such a shift was achieving a fully integrated health and well-being system between primary and secondary care, creating a foundational culture shift to embrace indigenous principles and values, overturning often severe health inequities, and empowering local communities and health consumers to reclaim the power, choice, and resources necessary to enable health and well-being.

We were privileged to be invited by the creators of this initiative to record, research and reflect on the governance processes of *Grassroots Health*—an entity encompassing government agencies, District Health Boards, primary care organizations, Kaupapa Māori organizations dedicated to health services, community and iwi—over the first two-and-a-half years of its early phases^1^. We should note that the initiative had begun 6 months prior to our engagement and hence our research does not include a discussion of its genesis, formation and start-up activities aside from the origin accounts that interviewees retrospectively narrated. The research invitation to us was extended by the core governing group at the point in time when the group had agreed, written and codified governing processes and were having to negotiate how those would be enacted between them. Therefore, the primary task for us in this research project was to document, with and for, the creators of the initiative (who were our collaborators rather than subjects), the way *Grassroots Health* negotiated and co-created appropriate effective governance practices for such a venture during the initial two-and-a-half-year period. To do so, we have chosen to theorize this through “the operational unfolding of paradox” lens (Clark, 2004, p. 174) and the identification of five core paradoxes. We take the “operational” of the above excerpt seriously realizing that “to observe is to make a distinction” (Clark, 2004, p. 177) and such distinctions create competing or observational frames. Therefore, it is important to say that the construction of these paradoxes is ours as researchers. Our collaborators certainly had awareness of some or all of these five paradoxes but they were not directly articulated to us as such. Given our accountability for such a construction (McClintock et al., 2003), we shared these with, and sought feedback from, our collaborators through the dissemination of transcripts and a pre-publication report. In response, our collaborators confirmed and endorsed these five paradoxes and they were subsequently “offered back” to the community via an interactive workshop on governance, policy and evaluation for cross-sector initiatives prior to the writing of this publication. In this way we moved between researcher-narrator and researcher-facilitator roles in a commitment to research *with*, or alongside, *Grassroots Health* (McClintock et al., 2003).

This article is structured in five parts. The first discusses governance and the recent proliferation of governance research beyond its traditional, mostly corporate, board concerns. The second focuses on the health context within which this initiative is located and particularly the international, national, and local trends and challenges which inform it closely; this is followed by a section detailing the research methodology. The case study findings are fourthly presented empirically using excerpts from interviews with the core members of *Grassroots Health*, structured through the previously mentioned five core paradoxes. We conclude with a series of recommendations that aim to speak directly, not to just those who research in this area, but those practitioners who are seeking to progress effective governance for similar kinds of social good initiatives. For this significant practitioner group we hope we create visibility of the early steps of embedding a coalition of core individuals and entities who can hold the unique trajectory and complexity of such ventures upon which so much of our future societal aspirations will depend.

Before continuing, however, it is necessary for us to acknowledge our positionality. We are a team of Pākehā (European) researchers from a range of disciplines^2^. In the Māori world, as Ritchie (1992, p. 51) has noted, we Pākehā are outsiders, visitors, and we will always be so. We come from a place of privilege, both within New Zealand and within the academy. Our research with the *Grassroots Health* partners was a result of their manaakitanga, their hospitality and generosity, their invitation to visit, record and interpret; and their critical feedback on the iterations of analysis we shared^3^. We also acknowledge that, while *Grassroots Health* was underpinned by a kaupapa Māori approach in that it centered Māori cultural aspirations, values and beliefs, as Pākehā researchers, our kaupapa (purpose) has been to let those involved speak for themselves. Our engagement with western understandings of adaptive governance as applied to these conversations, has been undertaken with the permission of our collaborators, as noted above. Our ongoing aspiration as researchers is to ensure we reciprocate the manaakitanga that has been shared with us, and to recognize that our understandings of Te Ao Māori will be always be incomplete (Ritchie, 1992; see also Pihama et al., 2015; Rauika, 2020).

## Rethinking Governance

Governance and its research are currently moving through a period of definitional ferment, divergence, and heterogeneity after decades of a largely corporate bias and an overwhelmingly regulatory focus. Ansell et al. (2016, p. 4–5) identified three major impacts on governance that account for this: firstly what they describe as “dramatic and turbulent” economic and societal change; secondly, “volatile and diffuse” changes to the immediate governance context; and thirdly, the tendency of problems and challenges to arrive at the “wicked” (as opposed to the technical and tame) end of a continuum. An associated shift is from what might be described as the authoritative command and control ethos to one of stewardship (Stoker, 2018) or governance as leadership (Chait et al., 2004) where collaborative, participatory and more inclusionary modes of working are required. Given the intersection of societal change, the nature of contemporary changes and the need for new governance practice (Ison and Straw, 2020), we are seeing calls for a systemic governance approach which highlights the importance of systems thinking, systemic co-inquiry and social learning (Ison et al., 2014; Ison, 2016, 2018)^4^. The cumulative effect of such shifts reinforces the predominant research view that greater complexity and the proliferation of wicked problems call for very different processes than the top-down, hierarchical and instrumental approaches that have been at the mainstream of governance, leadership, and management to date (Martin and Guarneros-Meza, 2013; Ansell et al., 2016; Innes and Booher, 2018).

Governance for social purpose could be considered a highly fragmented field. At the corporate end is research into the relationship between governance and corporate social responsibility which is orientated at understanding how corporates might balance economic and social imperatives and contribute to social projects without jeopardizing financial bottom lines (Sahut et al., 2019). At the social enterprise end is a focus on governance challenges such as shared accountability, conflicting agendas and interests, composition of boards, and issues around survival, growth, and independence (Ebrahim et al., 2014). In between, a plethora of alternative governance forms have emerged, including the ones central to this inquiry—these are collaborative and adaptive in orientation. It is important to note here that such emergence is not merely a matter of surface-level technologies and techniques but a fundamental redefinition of governance as relational. Ison et al. (2018) theorize such relationality by returning to governance’s etymological roots, “to steer,” highlighting the charting of a course through continually responding to uncertainty, (re)calibrating progress in response to contextual feedback, and re-negotiating purpose, or what they call “purpose elaborating” (Ison et al., 2018).

Such a steering metaphor harkens to the indigenous metaphor of wayfinding (Spiller et al., 2015) reminding us that traditional western concepts of governance have long been contested by indigenous scholars both in Aotearoa New Zealand and around the world. Processes of colonization imposed on indigenous peoples resulted in a model and practice of governance that has proved enduring in its harm. Moreover, as researchers we need to become more engaged with indigenous models of governance which are dynamic and involve consensual discussion, respectful deliberation, and are mana-enhancing^5^. What all share is a commitment to bringing *bottom up* and *top down* governance processes into more of a duality or even dialogue with each other.

Adaptive governance is mostly associated with environmental, ecological, and resource contexts although has begun to creep into other domains such as health, disaster and crisis, and law (Chaffin et al., 2014). It can be considered an often emergent response to the interconnected complexities of change, uncertainty, and dynamism that demand a wholesale systems thinking and acting involving stakeholders across all levels of the government, community and the private and professional sectors (Chaffin et al., 2014; Ison et al., 2014). It has a number of dimensions that intersect strongly with governance for social purpose. The first would be its often lengthy incubation where networks that have largely developed separately begin to coalesce; where “windows of opportunity” (Olsson et al., 2006) open up largely from crisis related or disruptive events that provide catalyzing impetus; and where informal configurations—sometimes called “shadow governance” (Lynch and Brunner, 2010) begin to take forms often in tandem with recognized authority structures.

The second would be in adaptive governance’s focus on bridging between micro conversations happening often at a local or community level and macro conversations happening in the government or institutional sphere (Brunner et al., 2005). Such a perspective redefines governance as a “pattern of practices” which demand sophisticated expertise and support for conversation, collaboration and conflict work (Brunner et al., 2005, p. 19). In a statement reminiscent of social purpose endeavors, Chaffin et al. (2014) noted that “community based initiatives often suffer from a lack of governing authority, legitimacy, funding, adequate flow of knowledge and resources, and sustained leadership” (p. 55), thus requiring diverse entities to work interdependently and converge resources and attention to overcome such lack. Adaptive governance offers a concept, process and set of practices by which such entities can come together to do exactly that.

Interdependence suggests the centrality of collaboration and participation which has become a focus in itself through what two academics refer to as “the fuzzy concept of collaborative governance” which encompasses a number of research streams including joined-up, network, interactive and participatory governance (Batory and Svensson, 2019a). Such governances are driven largely by the public administration and policy studies literatures and focus on initiatives that cross public and private spheres, thereby involving a broad diversity of stakeholders (Bingham and O’Leary, 2015). The tendency is to assume such governance is government led—something we will not assume at all for social purpose initiatives and which has pre-determined policy objectives—again something we will not assume for this study.

However, just as in adaptive governance, there are some lessons to learn for governance for social purpose. Waardenburg et al. (2020) identify three challenges in collaborative governance which they term *problem-solving*, *collaborative process*, and *multi-relational accountability* challenges that would appear to be exceedingly relevant to social purpose initiatives. The first, problem-solving, is the difficulty of even defining the core issue and its root causes and the political challenges of negotiating problem-definition and parameters. The second, challenges of collaborative process, refers to the need to work through different vested interests, values, cultures, and goals throughout the entire duration of the governance work. In the case of Aotearoa New Zealand, te Tiriti o Waitangi (the Treaty signed between Māori and the Crown)^6^ is a foundational document that requires that non-indigenous stakeholders and researchers recognize the specific roles, relationships and responsibilities that come with being partners in all governance arrangements.

The final challenge, multi-relational accountability, reflects the tensions and struggles about how to set responsibility, apportion accountabilities, and address performance issues. As one would expect, there is no straightforward resolution to such challenges, but the literatures clustering around collaborative governance both make them visible and normalize their occurrence. This brings the complexities of practice to the forefront of governance which has a tendency to focus on abstract models and frameworks. Our experience and expectation is that such challenges are inevitable in social purpose endeavors.

Such challenges, of course, are not new to governance. Indigenous governance reminds us, in fact, that such challenges are age old and not indexed to Western traditions and meta-narratives but deeply embedded in history and culture and, particularly, experiences of colonization, oppression, and racial dominance (Nikolakis et al., 2019; see also Hunt and Smith, 2006; Kukutai and Taylor, 2016; Cornell, 2018). Grey and Kuokkanen (2019, p. 8) defined governance with respect to indigenous self-determination where “governance is about a people choosing, collectively, how they organize themselves to run their own affairs and make decisions; share power, authority and responsibilities” and hence encompasses “the broader processes of which institutions are a part” ranging from “informal and localized decision-making processes to complex, centralized, formal structures.” Such self-governance sits at the very core of the social purpose initiative in Aotearoa New Zealand that was researched for this inquiry and, indeed, for any social purpose initiative (Joseph, 2005; Kahui and Richards, 2014).

Kamira (2007) argued that indigenous governance in Aotearoa New Zealand is complicated. While Kaitiakitanga as a concept centers around intergenerational stewardship, guardianship, collective responsibility, reciprocity and care, there are other critical principles that precede and inform kaitiakitanga, including mana (see text footnote 2) mana motuhake (independence, status, and sovereignty held by iwi), rangatiratanga (the hierarchical organization location of power and authority) and kawanatanga (political power and governance). Consequently there are deep cultural, historical, spiritual, and relational roots to the practice of governance in the Māori world. The 1840 Tiriti o Waitangi is a significant constitutional document that, when signed, was expected to support the sharing of governance between Māori and the Crown and enable indigenous self-determination and self-governance. In recent years, after over a century of breaches of its obligations, the Crown has re-engaged with the principles of equity, partnership, collective ownership, and protection. Te Tiriti o Waitangi is a living document and active framework whose principles drive any pursuit of social purpose and legitimizes shared or co-governance as the norm (Durie, 1998; Webster and Cheyne, 2017). Consequently, for non-indigenous stakeholders and researchers, it is necessary to recognize that Māori philosophies of governance are complex and connected to a worldview that understands land, life, spiritual essence, and connections across generations in a way that might not fit easily with Pākehā approaches. Māori emphasize collaboration, partnership and participation with the goal of bringing equity, inclusion and self-determination firmly into governance processes and purposes. It must be said that the ability to do this is very much a developing capacity for many stakeholders (including, and especially, the Crown) in the pursuit of social purpose that lives into the principles of Te Tiriti o Waitangi.

We would argue that any governance platform for social purpose will require dimensions from adaptive and systemic, collaborative and indigenous governance research and practice. We note that much of the research lies in the “gray literature” or the research driven by government agencies, private think-tanks and not-for-profit organizations, which brings a welcome practice and applied focus but lacks integration and connection with the research from purely academic institutions (Batory and Svensson, 2019b). What, therefore, remains underdeveloped is the theory–practice connection between these contemporary governance discourses and particularly the points of reference that provide any recognition of their negotiation. That is the purpose of this particular study. Therefore, our overarching research questions are: “*How does one negotiate the early phases of collaborative, grassroots or so-called ‘adaptive governance’ in the pursuit of social purpose outcomes?*” and “*What new governance practices will need to be forged and sustained in the pursuit of social purpose?*”

## Primary Health Care and Systemic Change

Through the Sustainable Development Goals (SDGs), the world has committed to an ambitious development agenda aimed at improving the health and well-being of all people (United Nations, 2015). The health-related SDGs, including goal 3, aimed at ensuring healthy lives and promoting well-being for all ages, can only be sustainably achieved with a stronger emphasis on Primary Health Care. Primary Health Care is a whole-of-society approach to health that aims equitably to maximize the level and distribution of health and well-being by focusing on people’s needs and meetings those needs as early as possible along the continuum from health promotion and disease prevention to treatment, rehabilitation and palliative care, and as close as feasible to people’s everyday environment. One of the underlying principles is that efforts to advance health and well-being are anchored in, and informed by, the community (World Health Organization (WHO) and the United Nations Children’s Fund (UNICEF), 2018).

As with integrated health and social care initiatives in countries across the world, the *Grassroots Health* initiative was guided by these international aspirations, aimed at overturning health inequities, and empowering local communities to achieve and health consumers to reclaim the power and resources necessary to enable health and well-being. There is a significant body of knowledge on such integrated care and service networks internationally and substantial literature on health and social care service partnerships that inform these initiatives. Most notable is information about unconventional health and care organizations affiliated with The King’s Fund in the United Kingdom. The King’s Fund report on integrated care systems (see for example, Charles, 2020) aimed at developing substantively different ways of supporting clients, and provide evidence that some organizations have been successful in delivering care with limited resources and providing effective support for people with complex needs.

The New Zealand government, as the context for the *Grassroots Health* initiative, has a commitment to improving access to primary health care within a devolved regional administrative-funding framework (Health and Disability System Review/Hauora Manaaki ki Aotearoa Whānui, 2020). The launch of the Primary Health Care Strategy (PHCS) in 2001 (King, 2001), followed by the establishment of primary health organizations (PHOs), set the direction and vision for primary health care services in New Zealand: delivery of better, sooner, more convenient (BSMC) services, expected integration of primary health and secondary care, an increased range of services in community settings, and greater collaboration to address prioritized vulnerable services, and achieve efficiencies. Several academics critiqued the strategy (e.g., Abel et al., 2005; Cumming, 2017), documenting compatibility of the PHCS with service delivery and the philosophy of care, but highlighting the challenges of implementing the PHCS. This is pertinent for the case study, since the *Grassroots Health* initiative was embedded in a community with a strong (30 per cent) Māori population (see Te Tai Tokerau Iwi Chief Executives Consortium, 2015). From 2016 onward, there have been some adjustments to the strategy and to funding of primary health care, with a documented commitment to making changes to address the complex (sometimes wicked) problems plaguing the health system (Health and Disability System Review/Hauora Manaaki ki Aotearoa Whānui, 2020; see also the Public Service Act, 2020). Nevertheless, the political environment and the administrative arrangements underpinning both access and funding remain complex, and this posed a number of governance challenges for *Grassroots Health*.

Delivery of health policy and services is underpinned by a long history of bureaucratic legacies that seldom fit contemporary local landscapes. As a result, there have been shifts at local levels in the way policies are designed and delivered, where principles of co-production, co-design, partnerships and collaboration across sectors inform outputs, outcomes, and practices (Larkin et al., 2015; Blomkamp, 2018). In New Zealand, such initiatives are still fledgling, and require funding, patience, and trust-rich relationships between stakeholders and communities. The ultimate goal is to be transformational in the way core services are delivered to communities and to create system-change along the way (Akama et al., 2019; Carroll et al., 2019; Maher, 2019).

Over the next 20 years, the health needs of the population in the Northland region of New Zealand will increase as a result of population growth and aging, and growing prevalence of long-term health conditions. A comparison with national socio-economic measures suggests that significant portions of the Northland region’s population are likely to experience hardship and deprivation (EHINZ, 2018), although such a comparison misses the significant community capacity, agency, resilience, and resourcefulness (Bishop et al., 2007). The forecasted future escalation in demand will mean services will need considerably increased capacity, but this cannot simply be *more of the same* if population outcomes are to improve, and inequities are to reduce (Te Tai Tokerau Iwi Chief Executives Consortium, 2015). The need for change is compounded by medium- to long-term forecasts of supply side constraints in operational and capital funding, and in the availability of workforce. Together, these factors point to the unsustainability of the health system in its current form in the Northland region. Future-proofing requires different resource-allocation patterns, and adoption of new ways of working that improve access, make better use of the available workforce, and improve service performance (Health and Disability System Review/Hauora Manaaki ki Aotearoa Whānui, 2020).

## Research Design: Case Study Approach

We have chosen a case study approach for “getting close to reality” (Flyvbjerg, 2006, p. 132). By this, we mean case studies are vehicles to work with the complexity and messiness of governance practice in a social purpose context and to discover how those unfold in “real life.” We note that a case study is “a choice of what is to be studied” (Stake, 2005, p. 443) or metaphorically, a “container” (Thomas, 2011, p. 12) for phenomena that “are in a constant interrelationship with one another” and “intermesh in myriad ways” (Thomas, 2011, p. 13). This closely parallels our understanding of governance presented earlier as dynamic, responsive, adaptive, relational, and responsive to uncertainty. The above means we have approached this case study, “not in the hope of proving anything, but rather in the hope of learning something” Flyvbjerg (2006, p. 224). What we hoped to learn were the practices required by those pursuing social purpose innovations through bespoke governance processes before formalized and tangible progress can be made visible. In short, we wanted access to the black-box (“internal complexities”) (Latour, 1999, p. 304) of governance practices that have been under-researched in governance to date—particularly governance initiatives pursuing complex social purpose endeavors. Exploring internal complexities means following a narrative sensibility or “hermeneutic composability” which seeks to ask “How does a sequence of events merge into a story? How are the elements woven together, if at all? What appears to depend on what? What contradicts? Where are there paradoxes?” (Thomas, 2010, p. 579). Such hermeneutic composability has shaped how we represent the case study where we resists the seduction of a cohesive, sequential narrative but rather offer five paradoxes—points of reference—in the negotiation of the early, often hidden stages of forming and building governance for social purpose.

**FIGURE 1:**
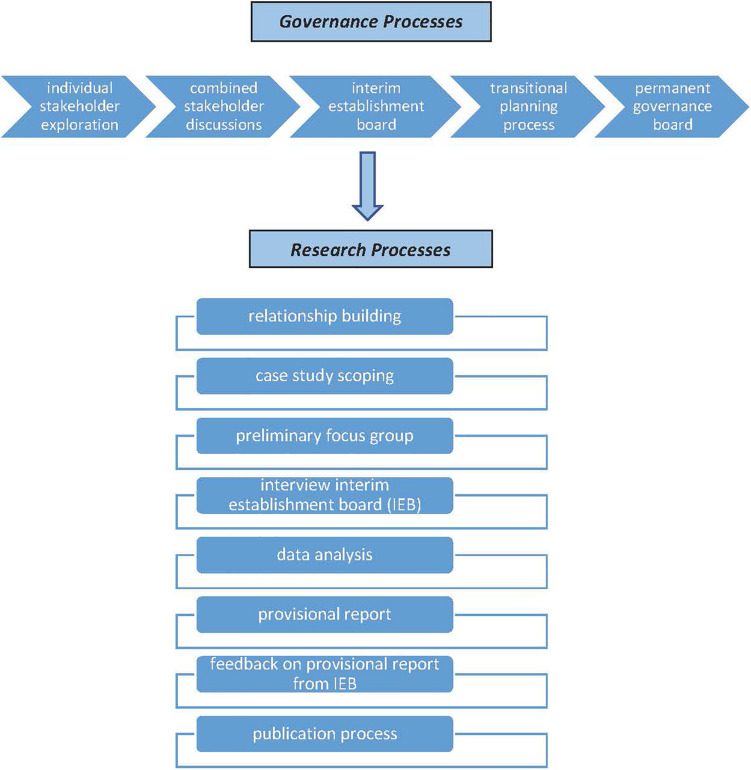
Intersection of grassroots health governance processes and research processes.

Engaging with *Grassroots Health*, as a possible case study, emerged from an exploratory strategic development opportunity undertaken by the three authors with the purpose of repositioning the university in service of capability and capacity building in high-growth areas of the country. We use the term *emerge* advisedly as the first phase of that work was centered on relationship building and was constituted through multiple meetings with different community, iwi and government agency leaders. Through such meetings, two possible capability and capacity building “projects” emerged and, while initial work began with both of them, *Grassroots Health* proved to have the stronger longevity and readiness for a research-led partnership approach. A case study process offered *Grassroots Health* the opportunity to reflect on their governance journey to date, the governance practices they were working through and, most importantly, share the learning with other, similar, initiatives throughout the country. For context, we set out in broad terms (to protect confidentiality) the overall trajectory of *Grassroots Health* and position this case study research relative to that in Figure 1.

Figure 1 illustrates that conversations and deliberations with the core stakeholders and contributors in *Grassroots Health* were predicated on building a positive, constructive relationships with the authors over time and a conscious shift from researching “on” a research subject toward researching “with” practitioners “within” their practice. As Pākēha documenting an initiative built from a kaupapa Māori approach, it was important for the research to be guided by indigenous principles, as outlined by Pe-Pua (2015). These include an awareness that social interactions between researchers and participants affect the quality of research data, and that participants, or contributors, are treated as equals within the research process. We also understood that our engagement needed to be appropriate to the norms of those with whom we were collaborating, and prioritize their language, words and accounts in our analysis (Te Awekōtuku, 1991; Smith, 1999; Pe-Pua, 2015). The relationship-building phase of our research encompassed six 2-h meetings with community, iwi, and government agency leaders and resulted in the opportunity to undertake the case study.

The empirical phase commenced with three focus groups, of 3–4 of the interim establishment board members at a time, to talk through the dates, documentation, history, and overview of the initiative. These sessions involved intense whiteboard sessions where the timeline and trajectory of the initiative were drawn and discussed. At such meetings we took notes, copied the whiteboard graphics and participated through asking questions and drawing out insights. This was followed with 12 in-depth interviews with each member of the interim establishment board including those who had participated in the focus group. We should note that this initiative had engaged with an intermediary entity to access mentoring services, project management and organizational/program development. This intermediary was considered core to the interim establishment board and was included in our empirical phase. The role of the intermediary is particularly important for this study as their role encompassed attention to effective governance practice and to sustaining overall movement and progress. To protect the anonymity of interviewees, we represent only their broad affiliations in Table 1.

**TABLE 1:** Interviewee affiliations.

**Affiliation**	**Number of interviews**
Community/Iwi Leaders	4
Senior Health Leaders	2
Community Health Organizations	4
Intermediary	2

Interviews ranged from 40 min (three interviews had to be conducted through phone) to 2 h and were semi-structured, ranging broadly around questions on the nature, trajectory and purpose of *Grassroots Health*, their own role in governance processes, critical moments and stakeholder relationships. We transcribed the recordings and each of the team members individually analyzed the material. The analysis followed an abductive process outlined by Thomas (2010, p. 579) of “questioning and surprise, intelligent noticing and serendipity” during which we gravitated firstly toward metaphor, narrative, symbols, myth, and contradictions and secondly toward the relationships and associations between them. We then met frequently as a group to discuss our (tentative) findings, converge our thinking and identify our findings. As noted previously, the construction of the five paradoxes came from these iterative rounds of analysis and conversation and particularly the coding clusters which highlighted seeming contradictions and oppositions. We re-focused on such clusters to arrive at the five core paradoxes we present here.

A report back to *Grassroots Health* was put together which outlined the paradoxes and sought feedback particularly for the findings and recommendations which follow the empirical section. The *Grassroots Health* participants pulled us toward more practical knowledge with their urgency that others needed the points of reference detailed here to gain both competence and confidence in what can feel like invisible, unrecognized and impossibly complex work. In this way, our contributors were centered within the research space as the experts with the knowledge and authority; they were with us from the outset, during data collection, our analysis and through to the completion of the report. Our hope is that this research will catalyze those committed to social purpose work and needing some research-based, theoretically informed practical knowledge with which to move forward.

## Empirical Material: A Storyline of Paradoxes

As mentioned earlier, we present this empirical section in the format of five paradoxes. Each paradox is constructed through the empirical material drawing on either a long-term narrative or a series of quotes from the interviews. We define paradoxes as competing frames or discourses which seem to contradict themselves, involve an “and/and” logic, or play on oppositions, but nonetheless represent some form of truth. Paradoxes flourish in contexts of complexity and adaptability and *Grassroots Health* represents exactly such a context given that it required:

• diverse entities to engage with each other
• a status quo breaking aspiration
• a non-linear pathway
• no certainty of resource, policy, or mandate
• a long-term trajectory but short-term deadlines
• the need to redistribute power and voice

### Paradox One: An Unchanging Pinnacle and Ever-Changing Conditions

“*[T]he tides are coming in and out, [but there is] the stability of the mana, manaia; because no matter what’s going down they’re always there.*

*The pinnacle doesn’t shift its axis that pinnacle still stays there. It’s there. It’s solid.*”

Paradox one is encapsulated by the metaphor above where the “*tides*” reflect the ever-changing conditions that need to be negotiated whilst the “*pinnacle*” reflects the overarching purpose which anchors the endeavor visibly and securely. “*Mana*” can be translated as power and authority and, in all the interviews the, source of mana was Te Tiriti o Waitangi and its promise of sovereignty (self-determination) and partnership for equity in health and well-being outcomes which required a fairer distribution of resource^7^. The *pinnacle*, across every single interview was something called a “kaupapa” which translates in multiple ways as a purpose or plan (Williams, 1997). Each of those images suggests something foundational in nature that provides a grounding, a core or direction. Hoskins and Jones (2017) indeed define kaupapa as precisely that: a foundation, set of principles and guide. It is the kaupapa, according to them, which sustains the action, directs attention to *why* to keep going when things get tough, offers clues on *how* to proceed when it is not clear and holds people to *what* matters the most at each stage of the endeavor.

The kaupapa constituting the “*pinnacle”* for Grassroots Health was the focus of conversations for approximately the first 6 months of the initial governance core group. It was written down and referred to at “*every meeting*” according to interviewees. A core task of the intermediary entity was to facilitate the creation of the kaupapa and to hold its centrality as the interim establishment board moved into the strategic and consultative/collaborative phase of its work with stakeholders. What seemed most striking to the research team was that every interviewee could speak to the kaupapa in close to identical terms when asked, demonstrating an impressive shared understanding of its meaning and a powerful connection to its place at the heart of the endeavor. Interviews themselves were full of unsolicited references to this kaupapa giving researchers the ability to see it live and in action. The kaupapa thus was continually embodied as a lived document, anchor and touchstone amidst the overall uncertainty and twists and turns as the way forward unfolded step by step—as the following narrative excerpt from one of the interviews evokes:

“*They’ll go^8^ : ‘You’ve got no idea what you’re doing then?’ I’ll say: ‘I’ve got the idea of knowing what I’m doing’. I’ve got behind me an end-to-end process to make it happen; what has to be done where, who we have to involve, what bits of this thing need to be sorted out to make it happen, and here’s the operating models for how it might operate. It’s all there, but … we have to do this bit first; we have to go to our communities and ask them, and then we’ll be able to work out how to best operate it and how you would do that. And, actually by the way, it’s not about you; it’s not about where your job is, because you aren’t relevant. What’s relevant is resolving the concerns of communities.*”

(Interviewee)

In this excerpt, what is “*relevant*” is the kaupapa and it supersedes every “*you*” and “*your job*” that is encountered during the “*end-to-end process to make it happen*.” Note the paradox in the need to “*go to our communities and ask them*” which is the call to partnership and to honor self-determination even whilst “*it’s all there*” seemingly ready to be implemented. But going back to the communities is also pragmatic, if we understand and recognize that knowledge and expertise sits within these communities, and if they are the actors charged with implementation. This is the “*tides*” (community priorities and voice) and “*pinnacle*” (kaupapa) in action.

### Paradox Two: Climbing Further and Turning Back

“*Let’s say we are trying to climb a mountain and the top was the final product. Like, every bit of a climb, you have to reach base camp, don’t you, every time you go up. At times, as you’re looking for your next base camp you’re going to come across crevasses and things where it gets icy. You don’t expect it to be just a straight walk; there’s going to be bits in between. I think we have continued to gain altitude slowly and surely. Just being determined to get to the top and had the expectation all the way along that it was going to be difficult. We didn’t even know what was coming ahead; what it would have been like going up. They’re* [stakeholders] *not sure what’s going to happen next, but they know where they want to get to and they’re actually going to have a rest or a break somewhere along the way. I think a lot of the times we’ve been over the hill and down the other side sort of thing, and having to come back and look over and watch people climbing*.”

This narrative of mountain climbing encapsulates the second paradox where movement forward into new terrain is accompanied by constant “back trips” to accompany and support those further behind who need “*a rest or a break somewhere along the way*.” Hence progression in such endeavors is back and forth, particularly for the interim establishment board who have to lead the challenge of forging new pathways into uncharted territory but shoulder the responsibility of getting others there as well. This narrative, of course, is resonant of paradox one where the “*pinnacle*,” once a beacon or stand-out landmark has now become a “*mountain*” that requires climbing. The climbing, in turn, is akin to negotiating “*tides*” where there is no “*straight walk*” but “*bits in between*” that evoke unpredictability (“*not sure what’s going to happen next*”) and uncertainty (“*we didn’t even know what was coming ahead*”).

This was just one narrative that talked of not leaving people behind, of not moving ahead too fast for others to engage, of being conscious of moving at the pace of others, of remembering to check whether others are keeping up, and of being prepared to pause, re-engage and halt movement. Getting to “*the top*” often gets most attention in challenging expeditions but “turning back” in these accounts proved equally, if not *more* vital, given the need to travel as a cross-sector partnership of diverse stakeholders the whole way.

### Paradox Three: Managing Process and Making Meaning

The third paradox is the contrast between two very different activities—managing process and making meaning—that needed to be done concurrently throughout. Each carries a very different point of focus, energy and set of practices as the following quotes attest:

Managing Process:

“*I’m very methodical and logical in my thinking too. I need to see a pathway A + B.*”

“*What I’m advocating for more, is greater efficiency through this; better planning and better outcomes.*”

“*If you don’t have enough goodwill you have to compensate operationally with almost perfect operational delivery at center and that takes time.*”

“*Underpinning this is a whole series of detailed work packages, detailed governance and approach to governance.*”

Making Meaning:

“*And, so I’m very careful, very careful, and maybe sometimes too careful and too cautious, that people need time sometimes to make the shift.*”

“*There’s creating the space and time for that; to have the maturity of thought, and develop consensus for disagreement, and that process. We’re so used to trying to push things, and it’s not informed discussion.*”

“*I don’t want a partial decision to move forward. I want everyone to move forward freely; because if we do, no-one can break that strength. No-one.*”

“*Generally*, *it’s as lack of engagement; I think we’re not asking people what matters to them.*”

As we can see, “managing process” is predicated on a discourse of efficiency, compensation, planning (either management or governance) and operations while “making meaning” prioritizes care, movement, engagement and shared decision making. The two together, of course, cannot be seen as incompatible as all complex endeavors require the significance that comes from meaning and the implementation that comes from process; however, the interviewees did talk to their interrelationship in a rare way. The intermediary held the planning, documentation, and process work as part of their project management role but such work was primarily referred to, by both themselves and others, as core to the transparency of the initiative—as visible cues or artifacts that would support stakeholders to have confidence in each other, and resources that could provide sites of re-engagement. As such, they played a key part in the broader narrative of making meaning and not, as could be inferred, run counter to it. The word “*underpinning*” points to the two as occupying a “two sides of a coin” logic where both need to be joined up but not necessarily front facing at the same time.

### Paradox Four: Building Trust and Seeking Conflict

Paradox 4 is another one of the paradoxes that has been well-recognized in the literature as, while seemingly at odds, it has a logic similar to a “chicken and egg” relationship where it is a single-direction, linear sequence—not straightforward. Key words and phrases are in bold to highlight the interrelationship between trust and conflict.

“*Trust isn’t some sort of intangible; it’s not based on being nice, it’s based on there being definitive truth.*”

“*It’s been really tough actually the experiences to date, but I think anything worthwhile is always hard. That’s just in the back of my mind, and in fact that’s a good sign.*”

“*Most of that work was about clearing the stuff, the misinformation away from the table; and that’s had to continue… throughout.*”

“*There’s agendas and there’s egos*.”

“*We had a couple of discussions where some hard issues were dealt with. I think it’s been the feature of this kaupapa mostly. You know how it is… when you address a hard issue, you don’t want to …, but then you do and transparency leads to peace in a way doesn’t it*….”

While interviewees varied in their tolerance of the degree of conflict present, most saw it as an inevitable component of the build-up and maintenance of trust, while a significant proportion credited it as the key that actually unlocked the trust and ensured it kept building. It is worth noting here that, within Te Ao Māori, conflict within decision making and negotiation processes is often anticipated and accepted. The opportunity for a place and space for different views to be heard, grievances aired and development of resolutions or pathways moving forward are all part of the ideal of kotahitanga (unity and consensus). Such conflict work could be understood as “accommodations of difference” (Russell and Ison, 2017) and associated, time and time again, with honesty, transparency, and truth which were considered essential to hold accountabilities across the stakeholders. Conflict work happened mostly outside of formal settings, in-between scheduled conversations and formed an ever-constant back-drop to the more strategic work. Stakeholders would seek each other out to work through differences, clarify discord, challenge assumptions, and confront intractable issues. No one pretended this was easy, some clearly felt uncomfortable at the extent of it, but many saw the capacity to engage in conflict productively as the single reason this endeavor made progress. It could be argued that trust was both the source and the outcome of this work with conflict which is implied in some of the above quotes where adjectives such as *tough* and *hard* are co-related with *worthwhile* and *peace*.

It is important to bring the tangibility of conflict into the social purpose and own there are as many “*agendas”* and “*egos*” there as anywhere else. The most telling insight is that conflict has been “*the feature of this kaupapa*” and was the challenge or difficulty that most interviewees raised to that question. Engaging courageously with conflict was seen as part and parcel of holding the kaupapa—if the kaupapa mattered, then anything that might prove an obstacle to realizing the kaupapa should be cleared. A number of interviewees talked about the astonishing trust that came from working through tensions and deep-seated struggles together at the same time as talking about the personal toll and cost in doing so but the weariness of having to do this again and again. How to build capacity and resilience for this trust/conflict work must be one of the biggest challenges for such governance.

### Paradox Five: Holding On (Power and Control) and Letting Go (Loss)

Complexities of power and control were sub-texts of all interviews. Not just the power and control of stakeholders (although those were present), but the entire system of power and control that pursuing social purpose can mobilize. Key to this were the dynamics of holding on of “power to” and the letting go of “power over” required, not just by the core governance participants, but by those they were seeking to influence and change. We should say from the outset that, at times, it was a key to hold on to what mattered and let go what did not and the judgment about which category things fell into was never clear cut. Hence it is too simplistic to see holding on as “bad” and letting go as “good” as it was understanding to which category things belonged that was key.

Holding On:

“*The control model is being where we’ve always been in health; that is not going to work.*”

“*They just want to know ‘how am I going to do it’, and ‘what’s my job going to be,’ and ‘is my job safe’, and ‘how important would I be in the structure’, or ‘how many people will I be controlling’. We’re saying, we’re not going to tell you any of that because we don’t know yet.*”

“*At times it gets lost in operational detail and competing interests and the like. You lose sight of it and you’re very likely to lose the value of the initiative I would suggest.*”

The quotes above speak to the propensity to hold health in a power and control frame economically, institutionally and socially and the understandable desire of stakeholders to keep their roles, jobs, and security in the face of uncertainty and change. Hold on to these two things and there is no transformation; be careless about how to loosen these and there is little trust or capacity to move. The tightrope of walking the two amidst “*operational detail and competing interests*” is helped by a different kind of holding on—holding on to the kaupapa, “*the value of the initiative*” and the purpose. *Grassroots Health* were adamant that kaupapa, value and purpose were the only things that should be held on to. Everything else had to be seen as malleable as the “letting go” quotes below show.

Letting Go:

“*For me I can give up control; I can give up form. I don’t care, as long as it works.*”

“*It’s the shifting of the paradigm and the balance of power, if you like, if you have to think of it in those terms, which you kind of do at this point. And that’s why our roles become immaterial because it’s the momentum of the model that will carry it.*”

“*I think everyone knows something is going to change, and everyone has to give something, and everyone’s going to lose something.*”

Many interviewees worked intentionally and reflexively on their capacity to let go personal or institutional/organizational power and control. Even where there was a commitment to do so, momentary failures of “letting go” were frequent and the source of much of the conflict work discussed earlier. Not surprisingly, relationships with power and control appear to remain an ongoing struggle for many who do this kind of work. These quotes all show a degree of acceptance for letting go and the losses that come with it. Notice that “*form*” and “*model*” appear enablers of generative letting go, again confirming that process, frameworks, and texts, such as the kaupapa have the capacity to aid these struggles. Loss has been theorized as inherent in adaptive work of any kind (Heifetz, 1994) where the capacity to see status quo imperatives as “*immaterial*” can create a space of action and possibility. Equally, the judgment as to what matters and what is ultimately immaterial, as our interviewees tell it, is not instant, not easy and never painless. In fact, that judgment goes to the core of how one works generatively with paradox which we explore in our findings and learnings section.

## Discussion: Responding to Paradox

In 1996, Ybema (1996, p. 40) argued that “paradoxes … seem to smile ironically at our nicely constructed theories with their clear-cut distinctions and point at an unthought-of-possibility.” The five paradoxes provide our answer to our first research question: “How does one negotiate the early phases of collaborative, grassroots or so-called “adaptive governance” in the pursuit of social purpose outcomes?” The “unthought-of-possibility” that our five empirical paradoxes point to is that the early stages of governance for social purpose—often still portrayed as formal, fixed and orientated at stability—are likely, in fact, to be emergent, precarious, mutable and conflictual. It is important to note here that those descriptors are not meant to apply to just less-than-effective governance experiences, but the norm. In short, one should anticipate and expect such governance configurations to follow a highly changeable, often circular, uncertain and uncomfortable trajectory and our contention in this article is that few are prepared for that, including governance participants themselves but also other stakeholders, funding and accreditation bodies and, of course, end users and clients. We offer these five paradoxes then as identifiable signposts or points of reference that those above can learn to recognize, navigate and grow capacity to work through.

Gaining such confidence for most will involve learning to live with paradox constructively. Indeed paradox has been increasingly of interest to organizational (Lewis, 2000) and governance scholars (Sundaramurthy and Lewis, 2003) as they catalyze a means to work powerfully with complexity, ambiguity, plurality and dynamism (Quinn and Cameron, 1988). Indeed any endeavors involving individuals, collectives and organizations are now seen, not only as “inherently paradoxical,” but usefully so, in that paradoxes “both hamper and encourage” the development of any change process (Lewis, 2000, p. 760). Lewis (2000), however, is also critical of using paradoxes as superficial clichés without inquiring deeply into their construction. She warns us about bringing an oppositional or bipolar logic to paradox (such as “trust is good/conflict is bad,” “forward movement is progress/backward movement is problematic”) and even a (seemingly positive) problem-solving logic in pursuit of resolving, clarifying or suppressing their tensions. Lewis (2000, p. 764) offers a paradox framework that would appear highly relevant to this study with three types of managing strategies: the first is acceptance (living with paradox); the second is confrontation (engaging with paradox); and the third is transcendence (intentionally thinking paradoxically). Our findings point to the need for those involved in social purpose governance to embrace transcendence and bring reflexivity, criticality, and creativity to the more analytic and strategic thinking modes associated with governance.

*Grassroots Health* could draw on considerable expertise and experience with transcending paradox. They contracted an intermediary entity whose practice is known for co-crafting a kaupapa, holding that kaupapa both internally and externally in the governance process on behalf of grassroots stakeholders particularly, calling out the responsibility of all to the kaupapa when required, and pursuing collective accountability through rigorous conflict and transparency processes. Indeed, one of our findings is that an intermediary is likely to be required to hold the paradoxes for both the process and the stakeholder group. While some governance configurations can support an intermediary function from within, many will need that function to come externally. The other powerful paradox expertise and experience came from indigenous (Māori) stakeholders, both internal and external, whose relationship with paradox goes back deeply into their ancestry and culture and who hold a comparable framework to the one we have outlined here through their facility with narrative, metaphor, myth, temporality, and symbolism.

The research on paradox warns us against eliminating paradoxes, as this may result in the oversimplifying of real and necessary tensions, thus leading the system to focus too narrowly on a limited number of too-instrumental goals or measures that cluster around only one of the ends of a paradox. The research tells us that both poles of a paradox should ideally be maintained at as minimal level as possible. At such a level, they retain their generative power by keeping the system “on its toes, in a state of continuous awareness of its own contradictions” and thus able to continually orientate to paradigm and system change (Clegg et al., 2002, p. 487). Thus a core finding from this research is that those in social purpose oriented governance need to protect these paradoxes, test their maintenance, and use this awareness to calibrate their ongoing work together.

We would note that, overall, the intensity of different ends of the paradoxes shifts over the trajectory of the governance process. We found that the right-hand side of the five paradoxes presented in this article—*a changing path, turning back, making meaning, seeking conflict*, and *letting go—*were the ones that represented a significant struggle in the early stages of governance formation. This provides a significant challenge for early stage governance given these would all be considered complex practices demanding a high degree of facilitative and participant cohesion and commitment. If these early stages of governance are successful, then the different stakeholders become more adept and practiced at these with possibly the need for an intermediary decreasing.

We note though, that if contested ends of the paradox are not held together in productive tension, an initiative risks either becoming status quo confirming and potentially complacent, or too divisive and confrontational to hold together thus threatening the paradigm and system change aspirations and redistribution of voice and power that social purpose initiatives require. With *Grassroots Health* there was a fear that moving away from the right-hand side (*a changing path, turning back, making meaning, seeking conflict, letting go*) too much into the relative comfort of the left-hand side (*an unchanging pinnacle, climbing further, managing process, building trust*, and *holding on*) would risk the radical nature of the transformation being sought. This fear seemed particularly salient as the process moved into its formal and permanent phase with a permanent trust governance board. The few who bridged both the interim establishment board and the permanent trust governance board were particularly aware of their need to act as internal intermediaries to protect both ends of the paradoxes.

Our final contribution is that paradigm change requires the non-linear movement that comes through moving between different ends of the paradoxes. This is not a comfortable or seamless rhythm and would be more akin to a series of pendulum swings than anything typifying flow or progression. Identifying such pendulum swings adds complexity given we know that there is a revolving door in participants entering and exiting board structures which was evident not only in the different make-up of the *Grassroots Health* interim establishment and permanent boards but also in the ongoing constitution of both of those over time. We suspect that most in this kind of governance are not prepared for such a bumpy, uneven, and discontinuous journey. Our overall contribution is that paradigm and system change require this kind of paradoxical capacity to create movement and momentum, whilst keeping all stakeholders involved and intact. Those in the next phase of *Grassroots Health* need to be mindful of the power of protecting paradoxes; losing sight of the ends and the tensions they bring is the biggest threat to the paradigm and system change aspirations of this endeavor.

In relation to our second research question, “What new governance practices will need to be forged and sustained in the pursuit of social purpose?” we offer the following recommendations in the spirit of speaking directly to governance and social purpose practitioners and offering something actionable:

1. Develop your kaupapa (set of foundational principles) early and use it to anchor conversations, planning, negotiations, and struggles. Have it written down, bring it to meetings, and evoke it often. All members of *Grassroots Health* could articulate their kaupapa and its meanings instantly in the interviews; it was palpably a shared and uniting force between them even as events unfolded in uncertainty and ambiguity (paradox 1).
2. Seek, or intentionally build, intermediary capability to hold movement forward and backwards and managing process and making meaning interdependently (paradoxes 2 and 3). The intermediary in *Grassroots Health* consciously managed the pace, focus, volume, and intensity of the overall process; something difficult to do for those stakeholders “in” the process as opposed to “on” the process.
3. Expect to spend as much time on relationships as the governance process itself particularly in the pursuit of building trust and seeking conflict and holding on and letting go (paradox 4). Members of *Grassroots Health* spent as much time on the phone, in face-to-face conversations, and in catch-ups as they did in formal procedures. Relationships that can persevere through conflict and capacity to talk to and move through power dynamics are the work in this early stages especially—not a distraction from it (paradox 4 and 5).
4. Managing process (documentation, protocols, and planning) is, perhaps somewhat counterintuitively, hugely important when embarking on endeavors high in ambiguity, complexity and uncertainty. While the latter demand agility, adaptability, and responsiveness more than compliance, the documentation, prototypes, and planning in *Grassroots Health* acted as decision points that stimulated the conversations needing to happen more than codes demanding automatic compliance (paradox 3).
5. Pay attention to the readiness of individuals and groups to take the next step and, if that readiness is not there yet, then wait for it to arrive without forcing it. Time moves differently when people are warming up to change and loss, as opposed to project milestones and deadlines. *Grassroots Health* core governance members referred to many such moments of pausing, regrouping, rethinking the pacing, and waiting for such readiness (paradox 2).
6. Inevitable to paradigm-breaking challenges is the requirement of all stakeholders to proactively engage with loss at some, if not multiple, parts of the process—whether that is loss of power, autonomy, resource, voice, or knowledge. Such losses will step up the need for the relationality, interdependence and care that has been constructed between stakeholders right from the beginning. *Grassroots Health* members highlighted such moments of loss as potentially critically risking connection and commitment (paradox 5).

## Conclusion

*Grassroots Health*, certainly to participants at the time, was experienced as rare and challenging, not only internally to itself, but to the broader policy context within which it sat. We contend, however, that the broader political ecosystem is no longer wholly hostile to paradoxical, legacy-disruptive ways of working. We should note that, in New Zealand, the government has been looking to break down system silos through a recent legislative initiatives (Public Service Act, 2020), and in its desire to see intergenerational well-being and cross-agency policy design become the norm. This suggests that the lessons learned from *Grassroots Health* may find fertile ground for emulation by others in our country but certainly globally as we all engage with a post-Covid-19 world. For those beginning on similar paradigm and systems change initiatives, practices powerfully developed by *Grassroots Health* should help in the navigation of complexity, contradictions and conflicts that define social purpose initiatives.

## Data Availability Statement

The raw data supporting the conclusions of this article will be made available by the authors, without undue reservation, to any qualified researcher.

## Ethics Statement

The studies involving human participants were reviewed and approved by the University of Auckland Human Ethics Committee. The participants provided their written informed consent to participate in this study.

## Author Contributions

BC collected the data, researched the governance section, led the data analysis, wrote the first draft, and finalized submission version. CF collected the data, collaborated in the data analysis, wrote the health section, and refined and edited the manuscript. JC contributed to the data analysis, researched and wrote the indigenous sections, and refined and edited the draft. All authors contributed to the article and approved the submitted version.

## Conflict of Interest

The authors declare that the research was conducted in the absence of any commercial or financial relationships that could be construed as a potential conflict of interest.

## Footnotes

1 *Kaupapa Māori* is a philosophical doctrine, incorporating the knowledge, skills, attitudes, and values of Māori society (Māori Dictionary, 2020) and, in practice, is concerned with Māori communities achieving cultural, educational, and social liberation, thereby supporting a process of decolonization (Akama et al., 2019).

2 *Pākehā* refers to a New Zealander of European descent.

3 *Manaakitanga* is defined as “hospitality, kindness, generosity, support—the process of showing respect, generosity, and care for others” (Māori Dictionary, 2020).

4 However, we acknowledge that these are “western” science or research paradigms and systems which cannot adequately account for Indigenous world views or Te Ao Māori concepts and practices of governance.

5 Mana is a critical principle in the Māori world, it underpins an understanding of governance from a Te Ao Māori perspective, and there is no single word or concept in English that captures the depth of its meaning. According to the Māori Dictionary (2020) it refers to “prestige, authority, control, power, influence, status, spiritual power, and charisma. Mana is also “a supernatural force in a person, place or object” and is inherited at birth. Mana comes with seniority and gives a person the authority to lead and to make decisions regarding social and political matters (Māori Dictionary, 2020).

6 The Treaty of Waitangi, New Zealand’s founding document, was meant to be a partnership between Māori and the British Crown. Although intended to create unity, mis-translation and misunderstandings on the part of the Crown resulted in two versions of the Treaty that, in turn, led to significant breaches of the Crown’s responsibilities to Māori over time (https://teara.govt.nz/en/treaty-of-waitangi).

7 *Rangatiratanga* is most often defined as chieftainship, and *tino-rangatiratanga* as full chieftainship. *Tino-rangatiratanga*, as it was used in the Treaty of Waitangi and interpreted today, has connotations of sovereignty, and of self-determination. *Kaitiakitanga*, associated with mana and rangatiratanga especially in the context of stewardship and governance of resources (Māori Dictionary, 2020).

8 “They” referred generically to who those engaged with *Grassroots Health* who were looking for certainty so can include any number of stakeholders including government agencies and funding providers.

## References

[B1] Abel S. Gibson D. Ehau T. Leach D. T. (2005). Implementing the primary health care strategy: a Māori health provider perspective. *Soc. Policy J. N. Z.* 25 70–87.

[B2] Akama Y. Hagen P. Whaanga-Schollum D. (2019). Problematizing replicable design to practice respectful, reciprocal, and relational co-designing with indigenous people. *Des. Cult.* 11 59–84. 10.1080/17547075.2019.1571306

[B3] Ansell C. Trondal J. Øgård M. (2016). *Governance in Turbulent Times.* London: Oxford University Press.

[B4] Batory A. Svensson S. (2019a). The fuzzy concept of collaborative governance: a systematic review of the state of the art. *Cent. Europ. J. Public. Policy* 13 28–39. 10.2478/cejpp-2019-0008

[B5] Batory A. Svensson S. (2019b). The use and abuse of participatory governance by populist governments. *Policy Polit* 47 227–244. 10.1332/030557319x15487805848586

[B6] Bingham L. B. O’Leary R. (eds) (2015). *Big Ideas in Collaborative Public Management.* London: Routledge.

[B7] Bishop R. Berryman M. Cavanagh T. Teddy L. (2007). *Te Kotahitanga Phase 3 Whanaungatanga: Establishing a Culturally Responsive Pedagogy of Relations in Mainstream Secondary School Classrooms.* Wellington, NZ: Ministry of Education.

[B8] Blomkamp E. (2018). The promise of co-design for public policy. *Aust. J. Publ. Admin.* 77 729–743. 10.1111/1467-8500.12310

[B9] Brunner R. D. Steelman T. A. Coe-Juell L. Cromley C. M. Edwards C. M. Tucker D. W. (2005). *Adaptive Governance: Integrating Science, Policy, and Decision Making.* New York, NY: Columbia University Press.

[B10] Carroll B. Fouché C. Curtin J. (2019). *Northland Primary Health Care Collaboration Kaupapa Initiative: A Case Narrative.* Auckland: University of Auckland.

[B11] Chaffin B. C. Gosnell H. Cosens B. A. (2014). A decade of adaptive governance scholarship: synthesis and future directions. *Ecol. Soc.* 19:56. 10.5751/ES-06824-190356 30174746

[B12] Chait R. Ryan W. Taylor B. (2004). *Governance as Leadership: Reframing the Work of Nonprofit Boards.* Hoboken, NJ: John Wiley & Sons Inc.

[B13] Charles A. (2020). *Integrated Care Systems Explained: Making Sense of Systems, Places and Neighbourhoods.* Avaliable at: https://www.kingsfund.org.uk/publications/integrated-care-systems-explained (accessed 10 August 2020).

[B14] Clark B. (2004). Paradox and the form of metamorphosis: systems theory in A Midsummer Night’s Dream. *Intertexts* 8 173–187.

[B15] Clegg S. de Cunha J. de Cunha M. (2002). Management paradoxes: a relational view. *Hum. Rel.* 55 483–503. 10.1177/0018726702555001

[B16] Cornell S. (2018). “Justice as position, justice as practice: indigenous governance at the boundary,” in *Indigenous Justice, Palgrave Socio-Legal Studies*, eds Hendry J. Tatum M. Jorgensen M. Howard-Wagner D. (London: Palgrave Macmillan), 11–26. 10.1057/978-1-137-60645-7_2

[B17] Cumming J. (2017). Health Policy. *Policy Quart.* 13 12–17.

[B18] Durie M. (1998). *Te Mana, Te Kāwanatanga. The Politics of Māori Self-Determination.* Melbourne, VIC: Oxford University Press.

[B19] Ebrahim E. Battilana J. Mair J. (2014). The governance of social enterprises: mission drift and accountability challenges in hybrid organizations. *Res. in Org. Behav.* 34 81–100. 10.1016/j.riob.2014.09.001

[B20] EHINZ, (2018). *Socioeconomic Deprivation Profile.* Wellington: Environmental Health Indicators New Zealand.

[B21] Flyvbjerg B. (2006). Five misunderstandings about case-study research. *Qual. Inq.* 12 219–245. 10.1177/1077800405284363

[B22] Grey S. Kuokkanen R. (2019). Indigenous governance of cultural heritage: searching for alternatives to co-management. *Int. J. Heritage Stud.* 25 1–23. 10.1080/13527258.2019.1703202

[B23] Health and Disability System Review/Hauora Manaaki ki Aotearoa Whānui, (2020). *Final Report/Pūrongo Whakamutunga.* Wellington: Ministry of Health.

[B24] Heifetz R. (1994). *Leadership Without Easy Answers.* Boston, MA: Harvard University Press.

[B25] Hoskins T. K. Jones A. (2017). *Critical Conversations in Kaupapa Māori.* Wellington: Huia Publishers.

[B26] Hunt J. Smith D. E. (2006). *Building Indigenous Community Governance in Australia: Preliminary Research Findings.* Working Paper No. 31/2006. Canberra, ACT: ANU Centre for Aboriginal Economic Policy Research.

[B27] Innes J. E. Booher D. E. (2018). *Planning With Complexity. An Introduction to Collaborative Rationality for Public Policy.* Abingdon: Routledge.

[B28] Ison R. Alexandra J. Wallis P. (2018). Governing in the Anthropocene: are there cyber-systemic antidotes to the malaise of modern governance? *Sust. Sci.* 33 1209–1223. 10.1007/s11625-018-0570-5 30220916 PMC6132393

[B29] Ison R. L. (2016). Governing in the Anthropocene: what future systems thinking in practice? *Syst. Res. Behav. Sci.* 33 595–613. 10.1002/sres.2421

[B30] Ison R. L. (2018). Governing the human-environment relationship: systemic practice. *Curr. Opin. Environ. Sust.* 33 114–123. 10.1016/j.cosust.2018.05.009

[B31] Ison R. L. Collins K. B. Wallis P. (2014). Institutionalising social learning: towards systemic and adaptive governance. *Environ. Sci. Policy* 53 105–117. 10.1016/j.envsci.2014.11.002

[B32] Ison R. L. Straw E. (2020). *The Hidden Power of Systems Thinking – Governance in a Climate Emergency.* Abingdon: Routledge.

[B33] Joseph R. (2005). *The Government of Themselves: Indigenous Peoples’ Internal Self-determination, Effective Self-governance and Authentic Representation - Waikato-Tainui, Ngāi Tahu and Nisga’a.* Doctor of Philosophy thesis, University of Waikato, Waikato.

[B34] Kahui V. Richards A. C. (2014). Lessons from resource management by indigenous Maori in New Zealand: governing the ecosystems as a commons. *Ecol. Econ.* 102 1–7. 10.1016/j.ecolecon.2014.03.006

[B35] Kamira R. (2007). “Kaitiakitanga and health informatics: introducing useful indigenous concepts of governance in the health sector,” in *Information Technology and Indigenous People*, eds Dyson L. E. Hendriks M. A. N. Grant S. (London: IGI Global), 31–50.

[B36] King A. (2001). *The Primary Health Care Strategy.* Wellington: Ministry of Health.

[B37] Kukutai T. Taylor J. (2016). *Indigenous Data Sovereignty. Toward an Agenda. Research Monograph No. 38.* Canberra, ACT: ANU Press.

[B38] Larkin M. Boden Z. V. R. Newton E. (2015). On the brink of genuinely collaborative care: experience-based co-design in mental health. *Qual. Health Res.* 25 1463–1476. 10.1177/1049732315576494 25829467

[B39] Latour B. (1999). *Pandora’s Hope. Essays on the Reality of Science Studies.* Cambridge, MA: Harvard University Press.

[B40] Lewis M. W. (2000). Exploring paradox: toward a more comprehensive guide. *Acad. Manag. Rev.* 25 760–776. 10.2307/259204

[B41] Lynch A. H. Brunner R. D. (2010). Learning from climate variability: adaptive governance and the Pacific ENSO applications center. *Weather Clim. Soc.* 2 311–319. 10.1175/2010WCAS1049.1

[B42] Maher L. (2019). *Six Tips on Co-design.* Avaliable at: http://koawatea.co.nz/six-tips-co-design/ (accessed 23 August 2020).

[B43] Māori Dictionary, (2020). *Māori Dictionary.* Avaliable at: www.maoridictionary.co.nz (accessed 23 August 2020).

[B44] Martin S. Guarneros-Meza V. (2013). Governing local partnerships: does external steering help local agencies address wicked problems? *Policy Polit.* 41 585–603. 10.1332/030557312X655819,

[B45] McClintock D. Ison R. Armson R. (2003). Metaphors for reflecting on research practice: researching with people. *J. Environ. PlanManag.* 46 715–731. 10.1080/0964056032000138454

[B46] Nikolakis W. Cornell S. Nelson H. W. Pierre S. Phillips G. (2019). *Reclaiming Indigenous Governance: Reflections and Insights from Australia, Canada, New Zealand, and the United States.* Tucson, AR: University of Arizona Press.

[B47] Olsson P. L. H. Gunderson S. R. Carpenter P. Ryan L. Lebel C. Folke C. (2006). Shooting the rapids: navigating transitions to adaptive governance of social-ecological systems. *Ecol. Soc.* 11 18–20.

[B48] Pe-Pua R. (2015). “Indigenous psychology,” in *International Encyclopedia of the Social & Behavioural Sciences*, ed. Wright J. D., (Oxford, UK: Oxford University Press), 788–794.

[B49] Pihama L. Tiakiwai S. Southey K. (eds) (2015). *Kaupapa Rangahau: A Reader. A Collection of Readings From the Kaupapa Rangahau Workshop Series.* Hamilton: University of Waikato.

[B50] Public Service Act, (2020). *Public Service Act.* Avaliable at: https://www.publicservice.govt.nz/our-work/reforms/public-service-reforms-factsheets/?e5920 = 5923-factsheet-1-overview-of-the-proposals (accessed 23 August 2020).

[B51] Quinn R. E. Cameron K. S. (eds) (1988). *Paradox and Transformation: Toward a Theory of Change in Organization and Management.* London: Harper & Row Publishers.

[B52] Rauika M. (2020). *A Guide to Vision Mātauranga: Lessons from Māori Voices in the New Zealand Science Sector.* Wellington: Rauika Mangai.

[B53] Ritchie J. (1992). *Becoming Bicultural.* Wellington: Huia Publishers.

[B54] Russell D. B. Ison R. L. (2017). Fruits of Gregory Bateson’s epistemological crisis: embodied mind-making and interactive experience in research and professional praxis. *Can. J. Commun.* 42 485–514.

[B55] Sahut J.-S. Peris-Ortiz M. Teulon F. (2019). Corporate social responsibility and governance. *J. Manag. Govern.* 23 901–912.

[B56] Smith L. (1999). *Decolonizing Methodologies: Research and Indigenous Peoples.* Dunedin: Zed Books.

[B57] Spiller C. Barclay-Kerr H. Panoho J. (2015). *Wayfinding Leadership: Ground Breaking Wisdom for Developing Leaders.* Wellington: Huia Publishing.

[B58] Stake R. E. (2005). “Qualitative case studies,” in *The Sage Handbook of Qualitative Research*, eds Denzin N. K. Lincoln Y. S., (London, UK: Sage Publications), 443–466.

[B59] Stoker G. (2018). Governance as theory: five propositions. *Int. Soc. Sci. J.* 50 27–28.

[B60] Sundaramurthy C. Lewis M. (2003). Control and collaboration: paradoxes of governance. *Acad. Manag. Rev.* 28 397–415. 10.2307/30040729

[B61] Te Awekōtuku N. (1991). *He Tikanga Whakaaro: Research Ethics in the Māori community.* Wellington: Ministry of Māori Affairs.

[B62] Te Tai Tokerau Iwi Chief Executives Consortium, (2015). *He Tangata, He Whenua, He Oranga.* Avaliable at: www.northlandnz.com/assets/Resource-Hub/Maori-Economy/2015-Tai-Tokerau-Maori-Growth-Strategy.pdf (accessed 23 August 2020).

[B63] Thomas G. (2010). Doing case study: abduction not induction; phronesis not theory. *Qual. Inq.* 16 575–582. 10.1177/1077800410372601

[B64] Thomas G. (2011). *How to Do Your Case Study: A Guide for Students and Researchers.* Thousand Oaks, CA: Sage Publications.

[B65] United Nations, (2015). *Transforming Our World: The 2030 Agenda for Sustainable Development. A/Res/70/1.* New York, NY: United Nations.

[B66] Waardenburg M. Groenleer M. de Jong J. Keijser B. (2020). Paradoxes of collaborative governance: investigating the real-life dynamics of multi-agency collaborations using a quasi-experimental action-research approach. *Public Manag. Rev.* 22 386–407. 10.1080/14719037.2019.1599056

[B67] Webster K. Cheyne C. (2017). Creating treaty-based local governance in New Zealand: māori and Pākehā views. *Kōtuitui NZ. J. Soc. Sci. Online* 12 146–164. 10.1080/1177083x.2017.1345766

[B68] Williams H. W. (1997). *A Dictionary of the Maori Language.* Wellington: G. P. Publications.

[B69] World Health Organization (WHO), and the United Nations Children’s Fund (UNICEF), (2018). *(A)Vision for Primary Health Care in the 21st Century: Towards Universal Health Coverage and The Sustainable Development Goals.* Geneva: WHO. (WHO/HIS/SDS/2018.X). Licence: CC BY-NC-SA 3.0 IGO.

[B70] Ybema S. (1996). “A duck-billed platypus in the theory and analysis of organizations: combinations of consensus and dissensus,” in *Contradictions in Context: Puzzling over Paradoxes in Contemporary Organizations*, eds Koot W. Sabelis I. Ybema S., (Amsterdam: VU University Press), 39–61.

